# 3-Carb­oxy-2-(2-cyclo­propyl­amino-4-methyl­pyridinium-3-yl­amino)­pyridinium dinitrate dihydrate

**DOI:** 10.1107/S1600536811040323

**Published:** 2011-10-08

**Authors:** Qiong Dong, Shouwen Jin, Kai Tong, Haidong He, YuanQi Yu

**Affiliations:** a Faculty of Science ZheJiang A & F University, Lin’An 311300, People’s Republic of China; b Tianmu College of ZheJiang A & F University, Lin’An 311300, People’s Republic of China

## Abstract

The two benzene rings in the cation of the title compound, C_15_H_18_N_4_O_2_^2+^·2NO_3_^−^·2H_2_O, are almost perpendicular [dihedral angle = 91.6 (2)°]. In the crystal, the components are linked by O—H⋯O, N—H⋯O and C—H⋯O hydrogen bonds.

## Related literature

For general background to hydrogen-bonding inter­actions, see: Lam & Mak (2000 ▶); Desiraju (2002 ▶); Liu *et al.* (2008 ▶); Biswas *et al.* (2009 ▶); Jin *et al.* (2010 ▶).
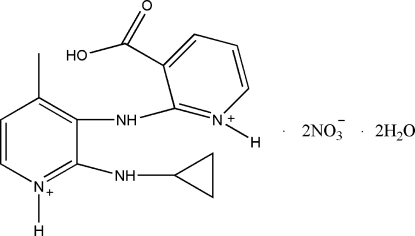


## Experimental

### 

#### Crystal data

C_15_H_18_N_4_O_2_^2+^·2NO_3_^−^·2H_2_O
*M* *_r_* = 446.39
Orthorhombic, 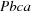

*a* = 7.4463 (6) Å
*b* = 15.0032 (14) Å
*c* = 35.975 (2) Å
*V* = 4019.0 (6) Å^3^
*Z* = 8
Mo *K*α radiation
μ = 0.13 mm^−1^
*T* = 298 K
0.44 × 0.36 × 0.34 mm

#### Data collection

Bruker SMART CCD diffractometer
Absorption correction: multi-scan (*SADABS*; Bruker, 2002 ▶) *T*_min_ = 0.947, *T*_max_ = 0.958
18931 measured reflections
3540 independent reflections
1964 reflections with *I* > 2σ(*I*)
*R*_int_ = 0.062

#### Refinement

*R*[*F* ^2^ > 2σ(*F* ^2^)] = 0.084
*wR*(*F* ^2^) = 0.295
*S* = 1.04
3540 reflections
281 parameters
H-atom parameters constrained
Δρ_max_ = 1.01 e Å^−3^
Δρ_min_ = −0.56 e Å^−3^

### 

Data collection: *SMART* (Bruker, 2002 ▶); cell refinement: *SAINT* (Bruker, 2002 ▶); data reduction: *SAINT*; program(s) used to solve structure: *SHELXS97* (Sheldrick, 2008 ▶); program(s) used to refine structure: *SHELXL97* (Sheldrick, 2008 ▶); molecular graphics: *SHELXTL* (Sheldrick, 2008 ▶); software used to prepare material for publication: *SHELXL97*.

## Supplementary Material

Crystal structure: contains datablock(s) global, I. DOI: 10.1107/S1600536811040323/jh2324sup1.cif

Structure factors: contains datablock(s) I. DOI: 10.1107/S1600536811040323/jh2324Isup2.hkl

Supplementary material file. DOI: 10.1107/S1600536811040323/jh2324Isup3.cml

Additional supplementary materials:  crystallographic information; 3D view; checkCIF report

## Figures and Tables

**Table 1:** Hydrogen-bond geometry (Å, °)

*D*—H⋯*A*	*D*—H	H⋯*A*	*D*⋯*A*	*D*—H⋯*A*
O10—H10*D*⋯O8^i^	0.85	2.10	2.942 (8)	171
O10—H10*C*⋯O4^ii^	0.85	2.19	3.037 (7)	172
O9—H9*D*⋯O5^iii^	0.85	2.55	3.089 (7)	123
O9—H9*D*⋯O4^iii^	0.85	2.07	2.916 (6)	174
O9—H9*C*⋯O1^iv^	0.85	1.96	2.809 (5)	173
O2—H2*A*⋯O9^v^	0.82	1.73	2.535 (5)	168
N4—H4⋯O7^vi^	0.86	1.94	2.787 (6)	166
N3—H3⋯O6	0.86	2.01	2.746 (6)	143
N2—H2⋯O3	0.86	1.98	2.810 (5)	163
N1—H1⋯O10	0.86	2.22	2.900 (6)	136
N1—H1⋯O1	0.86	2.05	2.697 (4)	131
C4—H4*A*⋯O5^vii^	0.93	2.34	3.162 (7)	147
C6—H6⋯O3^iv^	0.93	2.26	3.161 (6)	162
C11—H11⋯O8^vi^	0.93	2.57	3.252 (8)	131
C15—H15*A*⋯O3^ii^	0.96	2.58	3.533 (7)	174

Symmetry codes: (i) 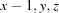; (ii) 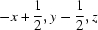; (iii) 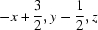; (iv) 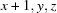; (v) 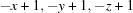; (vi) 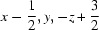; (vii) 
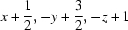
.

**Fig. 1.**
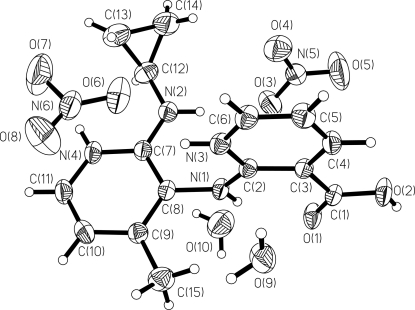
The structure of the title compound, showing the atom-numbering scheme. Displacement ellipsoids are drawn at the 30% probability level.

**Fig. 2.**
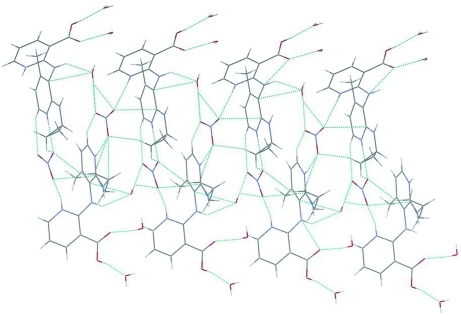
The one-dimensional doublechain formed through CH—O, and CH2—O interactions running along the a axis direction.

## supplementary crystallographic information

### Comment

Intermolecular interactions are responsible for crystal packing and gaining an understanding of them allows us to comprehend collective properties and permits the design of new crystals with specific physical and chemical properties (Lam & Mak, 2000). Hydrogen bonding is one of the most important noncovalent interactions that determines and controls the assembly of molecules and ions (Desiraju, 2002, Liu *et al.*, 2008, Biswas *et al.*, 2009).

Organic salts based on hydrogen bonding are also a research field receiving great attention in recent years. As an extension of our study concentrating on hydrogen bonded assembly of organic acid and organic base (Jin *et al.*, 2010), herein we report the crystal structure of 2-(2-(cyclopropylamino)-4-methylpyridinium-3-ylamino) nicotinic acid dinitrate dihydrate.

The crystal of the title compound of the formula C_15_H_22_N_6_O_10_ was obtained by recrystallization of 2-(2-(cyclopropylamino)-4-methylpyridin-3-ylamino) nicotinic acid from aqueous solution of HNO_3_.

The asymmetric unit of the compound consists of one dication, two nitrate anions, and two free water molecules (Fig. 1), respectively.

The compound is an organic salt. At every cation there are bound two nitrate anions through the N—H···O hydrogen bond. And the two water molecules were connected with carbonyl and OH of the carboxyl group of the cation rspectively *via* the O—H···O hydrogen bond. Under these interactions the cation, the anions, and the water molecules form a pentacomponent adduct. Such kind of adducts were connected together by the N—H···O, O—H···O, O-pi, and CH_2_—O interactions to form a one-dimensional chain running along the *a* axis direction. Two such chains were joined together *via* the CH—O, and CH_2_—O interactions to form double chain structure (Fig. 2). The double chains were linked together *via* the water molecule that is bound with the carboxyl group to form two-dimensional sheet extending along the *ac* plane. The two-dimensional sheets further stacked along the *b* axis direction through the nitrate group to form three-dimensional layer network structure.

### Experimental

A solution of 2-(2-(cyclopropylamino)-4-methylpyridin-3-ylamino) nicotinic acid (28.4 mg, 0.1 mmol) was dissolved in 5 ml of water and 1 ml of conc. HNO_3_ under continuous stirring. The solution was stirred for about 1 h at room temperature, then the solution was filtered into a test tube. The solution was left standing at room temperature for several days, colorless block crystals were isolated after slow evaporation of the solution in air at ambient temperature. The crystals were collected and dried in air to give the title compound.

### Refinement

Hydrogen atoms attached to the C atoms were placed in calculated positions with d(C—H) = 0.93–0.97 Å. Positions of the hydrogen atoms at the NH, OH, and COOH groups were located from the Fourier difference syntheses and refined independently. All *U*_iso_ values were restrained on *U*_eq_ values of the parent atoms.

### Crystal data

**Table:**

C_15_H_18_N_4_O_2_^2+^·2NO_3_^−^·2H_2_O	*D*_x_ = 1.475 Mg m^−^^3^
*M**_r_* = 446.39	Mo *K*α radiation, λ = 0.71073 Å
Orthorhombic, *P**b**c**a*	Cell parameters from 887 reflections
*a* = 7.4463 (6) Å	θ = 2.7–20.4°
*b* = 15.0032 (14) Å	µ = 0.13 mm^−^^1^
*c* = 35.975 (2) Å	*T* = 298 K
*V* = 4019.0 (6) Å^3^	Block, colorless
*Z* = 8	0.44 × 0.36 × 0.34 mm
*F*(000) = 1872	

### Data collection

**Table:**

Bruker SMART CCD diffractometer	3540 independent reflections
Radiation source: fine-focus sealed tube	1964 reflections with *I* > 2σ(*I*)
graphite	*R*_int_ = 0.062
φ and ω scans	θ_max_ = 25.0°, θ_min_ = 2.7°
Absorption correction: multi-scan (*SADABS*; Bruker, 2002)	*h* = −8→8
*T*_min_ = 0.947, *T*_max_ = 0.958	*k* = −17→17
18931 measured reflections	*l* = −33→42

### Refinement

**Table:**

Refinement on *F*^2^	Primary atom site location: structure-invariant direct methods
Least-squares matrix: full	Secondary atom site location: difference Fourier map
*R*[*F*^2^ > 2σ(*F*^2^)] = 0.084	Hydrogen site location: inferred from neighbouring sites
*wR*(*F*^2^) = 0.295	H-atom parameters constrained
*S* = 1.04	*w* = 1/[σ^2^(*F*_o_^2^) + (0.1827*P*)^2^] where *P* = (*F*_o_^2^ + 2*F*_c_^2^)/3
3540 reflections	(Δ/σ)_max_ = 0.001
281 parameters	Δρ_max_ = 1.01 e Å^−^^3^
0 restraints	Δρ_min_ = −0.56 e Å^−^^3^

### Special details

**Table:**

Geometry. All e.s.d.'s (except the e.s.d. in the dihedral angle between two l.s. planes) are estimated using the full covariance matrix. The cell e.s.d.'s are taken into account individually in the estimation of e.s.d.'s in distances, angles and torsion angles; correlations between e.s.d.'s in cell parameters are only used when they are defined by crystal symmetry. An approximate (isotropic) treatment of cell e.s.d.'s is used for estimating e.s.d.'s involving l.s. planes.
Refinement. Refinement of *F*^2^ against ALL reflections. The weighted *R*-factor *wR* and goodness of fit *S* are based on *F*^2^, conventional *R*-factors *R* are based on *F*, with *F* set to zero for negative *F*^2^. The threshold expression of *F*^2^ > σ(*F*^2^) is used only for calculating *R*-factors(gt) *etc*. and is not relevant to the choice of reflections for refinement. *R*-factors based on *F*^2^ are statistically about twice as large as those based on *F*, and *R*- factors based on ALL data will be even larger.

### Fractional atomic coordinates and isotropic or equivalent isotropic displacement parameters (Å^2^)

**Table:**

	*x*	*y*	*z*	*U*_iso_*/*U*_eq_	
N1	0.4596 (5)	0.5310 (2)	0.60657 (9)	0.0449 (9)	
H1	0.3541	0.5202	0.5979	0.054*	
N2	0.3878 (5)	0.6437 (2)	0.66673 (11)	0.0551 (11)	
H2	0.3653	0.6592	0.6442	0.066*	
N3	0.7403 (5)	0.5937 (2)	0.59845 (10)	0.0467 (9)	
H3	0.7653	0.5784	0.6209	0.056*	
N4	0.4826 (5)	0.5354 (3)	0.70757 (10)	0.0529 (10)	
H4	0.4567	0.5709	0.7256	0.064*	
N5	0.3305 (7)	0.7673 (3)	0.58219 (12)	0.0604 (11)	
N6	0.9273 (8)	0.5859 (4)	0.69535 (14)	0.0758 (14)	
O1	0.2512 (4)	0.5323 (2)	0.54524 (9)	0.0611 (10)	
O2	0.3557 (5)	0.6001 (2)	0.49541 (8)	0.0666 (10)	
H2A	0.2583	0.5853	0.4868	0.100*	
O3	0.2495 (5)	0.7115 (3)	0.59976 (12)	0.0820 (13)	
O4	0.4801 (6)	0.7916 (3)	0.59228 (13)	0.0889 (13)	
O5	0.2641 (9)	0.7961 (4)	0.55453 (15)	0.143 (2)	
O6	0.8651 (6)	0.6237 (4)	0.66917 (13)	0.1026 (15)	
O7	0.8916 (9)	0.6246 (4)	0.72714 (14)	0.139 (2)	
O8	1.0224 (9)	0.5242 (4)	0.69928 (17)	0.128 (2)	
O9	0.9337 (5)	0.4322 (3)	0.53919 (11)	0.0792 (12)	
H9C	1.0258	0.4656	0.5397	0.095*	
H9D	0.9505	0.3895	0.5543	0.095*	
O10	0.0912 (7)	0.4787 (3)	0.62102 (13)	0.1114 (17)	
H10C	0.0673	0.4250	0.6152	0.134*	
H10D	0.0670	0.4862	0.6439	0.134*	
C1	0.3673 (6)	0.5733 (3)	0.52926 (12)	0.0470 (11)	
C2	0.5772 (6)	0.5728 (3)	0.58415 (11)	0.0397 (10)	
C3	0.5404 (6)	0.5972 (3)	0.54734 (11)	0.0417 (10)	
C4	0.6701 (6)	0.6429 (3)	0.52733 (12)	0.0502 (11)	
H4A	0.6464	0.6598	0.5030	0.060*	
C5	0.8353 (7)	0.6638 (3)	0.54316 (13)	0.0564 (12)	
H5	0.9221	0.6947	0.5298	0.068*	
C6	0.8655 (6)	0.6383 (3)	0.57803 (13)	0.0529 (12)	
H6	0.9758	0.6514	0.5889	0.063*	
C7	0.4545 (6)	0.5623 (3)	0.67250 (12)	0.0474 (11)	
C8	0.4974 (6)	0.5038 (3)	0.64316 (11)	0.0424 (10)	
C9	0.5662 (6)	0.4192 (3)	0.65037 (13)	0.0492 (12)	
C10	0.5932 (7)	0.3961 (3)	0.68769 (15)	0.0611 (14)	
H10	0.6408	0.3405	0.6935	0.073*	
C11	0.5509 (7)	0.4537 (4)	0.71544 (14)	0.0612 (14)	
H11	0.5688	0.4370	0.7401	0.073*	
C12	0.3515 (8)	0.7062 (3)	0.69516 (15)	0.0675 (15)	
H12	0.2439	0.6942	0.7100	0.081*	
C13	0.4961 (10)	0.7496 (4)	0.7146 (2)	0.0862 (19)	
H13A	0.6180	0.7342	0.7076	0.103*	
H13B	0.4794	0.7625	0.7408	0.103*	
C14	0.3883 (10)	0.7997 (4)	0.6879 (2)	0.091 (2)	
H14A	0.3044	0.8434	0.6975	0.110*	
H14B	0.4431	0.8151	0.6643	0.110*	
C15	0.6116 (8)	0.3554 (4)	0.62113 (16)	0.0719 (16)	
H15A	0.5122	0.3158	0.6172	0.108*	
H15B	0.7152	0.3216	0.6285	0.108*	
H15C	0.6374	0.3869	0.5985	0.108*	

### Atomic displacement parameters (Å^2^)

**Table:**

	*U*^11^	*U*^22^	*U*^33^	*U*^12^	*U*^13^	*U*^23^
N1	0.042 (2)	0.058 (2)	0.0342 (19)	−0.0082 (18)	−0.0056 (16)	0.0035 (17)
N2	0.067 (3)	0.053 (2)	0.045 (2)	0.005 (2)	−0.0016 (19)	−0.0010 (18)
N3	0.041 (2)	0.053 (2)	0.046 (2)	−0.0064 (17)	−0.0023 (17)	−0.0017 (17)
N4	0.059 (3)	0.065 (3)	0.035 (2)	−0.003 (2)	−0.0017 (18)	0.0049 (19)
N5	0.066 (3)	0.058 (2)	0.058 (3)	−0.004 (2)	−0.005 (2)	0.005 (2)
N6	0.077 (4)	0.097 (4)	0.054 (3)	−0.022 (3)	−0.015 (3)	0.007 (3)
O1	0.051 (2)	0.088 (2)	0.0446 (18)	−0.0183 (18)	−0.0130 (16)	0.0122 (17)
O2	0.065 (2)	0.097 (3)	0.0374 (19)	−0.0155 (19)	−0.0153 (16)	0.0130 (17)
O3	0.072 (3)	0.076 (2)	0.098 (3)	−0.018 (2)	−0.026 (2)	0.032 (2)
O4	0.077 (3)	0.097 (3)	0.092 (3)	−0.028 (2)	−0.005 (2)	−0.003 (2)
O5	0.155 (6)	0.173 (5)	0.102 (4)	−0.018 (4)	−0.050 (4)	0.071 (4)
O6	0.100 (4)	0.142 (4)	0.066 (3)	0.002 (3)	−0.023 (3)	0.007 (3)
O7	0.174 (6)	0.179 (5)	0.064 (3)	0.041 (5)	−0.020 (3)	−0.015 (3)
O8	0.143 (5)	0.115 (4)	0.126 (5)	0.025 (4)	0.030 (4)	0.040 (4)
O9	0.076 (3)	0.079 (2)	0.083 (3)	−0.015 (2)	−0.037 (2)	0.016 (2)
O10	0.128 (4)	0.121 (4)	0.085 (3)	−0.014 (3)	0.004 (3)	−0.013 (3)
C1	0.050 (3)	0.057 (3)	0.034 (2)	−0.002 (2)	−0.005 (2)	−0.001 (2)
C2	0.037 (2)	0.044 (2)	0.037 (2)	−0.0040 (19)	−0.0001 (18)	−0.0010 (18)
C3	0.042 (2)	0.046 (2)	0.037 (2)	−0.0034 (19)	−0.0008 (19)	−0.0001 (19)
C4	0.053 (3)	0.059 (3)	0.039 (2)	−0.006 (2)	−0.001 (2)	0.007 (2)
C5	0.049 (3)	0.064 (3)	0.056 (3)	−0.010 (2)	0.006 (2)	0.006 (2)
C6	0.040 (3)	0.064 (3)	0.055 (3)	−0.009 (2)	−0.005 (2)	−0.002 (2)
C7	0.051 (3)	0.051 (3)	0.040 (3)	−0.003 (2)	−0.003 (2)	0.002 (2)
C8	0.040 (2)	0.052 (3)	0.035 (2)	−0.006 (2)	−0.0019 (19)	0.0020 (19)
C9	0.052 (3)	0.050 (3)	0.046 (3)	−0.004 (2)	−0.001 (2)	0.002 (2)
C10	0.064 (3)	0.056 (3)	0.062 (3)	0.009 (2)	−0.008 (3)	0.014 (3)
C11	0.072 (4)	0.070 (3)	0.041 (3)	−0.001 (3)	−0.005 (2)	0.013 (3)
C12	0.069 (4)	0.067 (3)	0.066 (3)	0.001 (3)	0.003 (3)	−0.015 (3)
C13	0.091 (5)	0.074 (4)	0.093 (5)	0.000 (3)	−0.007 (4)	−0.022 (3)
C14	0.113 (6)	0.067 (4)	0.095 (5)	0.019 (4)	−0.011 (4)	−0.018 (3)
C15	0.080 (4)	0.067 (3)	0.069 (4)	0.006 (3)	0.011 (3)	−0.008 (3)

### Geometric parameters (Å, °)

**Table:**

N1—C2	1.346 (5)	C2—C3	1.401 (6)
N1—C8	1.406 (5)	C3—C4	1.386 (6)
N1—H1	0.8600	C4—C5	1.392 (7)
N2—C7	1.335 (6)	C4—H4A	0.9300
N2—C12	1.414 (6)	C5—C6	1.331 (6)
N2—H2	0.8600	C5—H5	0.9300
N3—C2	1.356 (5)	C6—H6	0.9300
N3—C6	1.363 (6)	C7—C8	1.410 (6)
N3—H3	0.8600	C8—C9	1.393 (6)
N4—C7	1.341 (5)	C9—C10	1.401 (7)
N4—C11	1.358 (6)	C9—C15	1.462 (7)
N4—H4	0.8600	C10—C11	1.357 (7)
N5—O5	1.192 (6)	C10—H10	0.9300
N5—O3	1.210 (5)	C11—H11	0.9300
N5—O4	1.227 (6)	C12—C13	1.440 (8)
N6—O8	1.174 (7)	C12—C14	1.452 (8)
N6—O6	1.193 (6)	C12—H12	0.9800
N6—O7	1.310 (7)	C13—C14	1.460 (9)
O1—C1	1.207 (5)	C13—H13A	0.9700
O2—C1	1.285 (5)	C13—H13B	0.9700
O2—H2A	0.8200	C14—H14A	0.9700
O9—H9C	0.8500	C14—H14B	0.9700
O9—H9D	0.8501	C15—H15A	0.9600
O10—H10C	0.8499	C15—H15B	0.9600
O10—H10D	0.8501	C15—H15C	0.9600
C1—C3	1.488 (6)		
			
C2—N1—C8	124.4 (4)	N2—C7—N4	118.7 (4)
C2—N1—H1	117.8	N2—C7—C8	122.5 (4)
C8—N1—H1	117.8	N4—C7—C8	118.8 (4)
C7—N2—C12	124.4 (4)	C9—C8—N1	120.8 (4)
C7—N2—H2	117.8	C9—C8—C7	120.7 (4)
C12—N2—H2	117.8	N1—C8—C7	118.4 (4)
C2—N3—C6	121.5 (4)	C8—C9—C10	117.2 (4)
C2—N3—H3	119.3	C8—C9—C15	123.2 (4)
C6—N3—H3	119.3	C10—C9—C15	119.6 (5)
C7—N4—C11	121.8 (4)	C11—C10—C9	120.9 (5)
C7—N4—H4	119.1	C11—C10—H10	119.5
C11—N4—H4	119.1	C9—C10—H10	119.5
O5—N5—O3	118.6 (5)	C10—C11—N4	120.6 (4)
O5—N5—O4	121.1 (5)	C10—C11—H11	119.7
O3—N5—O4	120.3 (5)	N4—C11—H11	119.7
O8—N6—O6	134.8 (7)	N2—C12—C13	120.6 (5)
O8—N6—O7	111.5 (6)	N2—C12—C14	118.3 (5)
O6—N6—O7	113.5 (6)	C13—C12—C14	60.6 (4)
C1—O2—H2A	109.5	N2—C12—H12	115.4
H9C—O9—H9D	108.2	C13—C12—H12	115.4
H10C—O10—H10D	108.6	C14—C12—H12	115.4
O1—C1—O2	124.3 (4)	C12—C13—C14	60.1 (4)
O1—C1—C3	122.4 (4)	C12—C13—H13A	117.8
O2—C1—C3	113.4 (4)	C14—C13—H13A	117.8
N1—C2—N3	117.6 (4)	C12—C13—H13B	117.8
N1—C2—C3	124.1 (4)	C14—C13—H13B	117.8
N3—C2—C3	118.3 (4)	H13A—C13—H13B	114.9
C4—C3—C2	118.9 (4)	C12—C14—C13	59.3 (4)
C4—C3—C1	119.7 (4)	C12—C14—H14A	117.8
C2—C3—C1	121.3 (4)	C13—C14—H14A	117.8
C3—C4—C5	121.0 (4)	C12—C14—H14B	117.8
C3—C4—H4A	119.5	C13—C14—H14B	117.8
C5—C4—H4A	119.5	H14A—C14—H14B	115.0
C6—C5—C4	118.1 (4)	C9—C15—H15A	109.5
C6—C5—H5	121.0	C9—C15—H15B	109.5
C4—C5—H5	121.0	H15A—C15—H15B	109.5
C5—C6—N3	122.3 (4)	C9—C15—H15C	109.5
C5—C6—H6	118.9	H15A—C15—H15C	109.5
N3—C6—H6	118.9	H15B—C15—H15C	109.5
			
C8—N1—C2—N3	−3.2 (6)	C11—N4—C7—C8	−0.1 (7)
C8—N1—C2—C3	178.0 (4)	C2—N1—C8—C9	−90.6 (5)
C6—N3—C2—N1	−178.0 (4)	C2—N1—C8—C7	93.0 (5)
C6—N3—C2—C3	0.9 (6)	N2—C7—C8—C9	−179.7 (4)
N1—C2—C3—C4	177.7 (4)	N4—C7—C8—C9	0.6 (7)
N3—C2—C3—C4	−1.1 (6)	N2—C7—C8—N1	−3.3 (6)
N1—C2—C3—C1	−3.7 (6)	N4—C7—C8—N1	177.0 (4)
N3—C2—C3—C1	177.5 (4)	N1—C8—C9—C10	−177.4 (4)
O1—C1—C3—C4	178.8 (4)	C7—C8—C9—C10	−1.1 (7)
O2—C1—C3—C4	−1.0 (6)	N1—C8—C9—C15	3.1 (7)
O1—C1—C3—C2	0.2 (7)	C7—C8—C9—C15	179.4 (5)
O2—C1—C3—C2	−179.5 (4)	C8—C9—C10—C11	1.1 (7)
C2—C3—C4—C5	0.5 (7)	C15—C9—C10—C11	−179.5 (5)
C1—C3—C4—C5	−178.0 (4)	C9—C10—C11—N4	−0.5 (8)
C3—C4—C5—C6	0.3 (7)	C7—N4—C11—C10	0.0 (8)
C4—C5—C6—N3	−0.6 (7)	C7—N2—C12—C13	71.3 (7)
C2—N3—C6—C5	0.0 (7)	C7—N2—C12—C14	142.1 (6)
C12—N2—C7—N4	3.0 (7)	N2—C12—C13—C14	107.4 (6)
C12—N2—C7—C8	−176.6 (5)	N2—C12—C14—C13	−111.1 (6)
C11—N4—C7—N2	−179.7 (4)		

### Hydrogen-bond geometry (Å, °)

**Table:**

*D*—H···*A*	*D*—H	H···*A*	*D*···*A*	*D*—H···*A*
O10—H10D···O8^i^	0.85	2.10	2.942 (8)	171.
O10—H10C···O4^ii^	0.85	2.19	3.037 (7)	172.
O9—H9D···O5^iii^	0.85	2.55	3.089 (7)	123.
O9—H9D···O4^iii^	0.85	2.07	2.916 (6)	174.
O9—H9C···O1^iv^	0.85	1.96	2.809 (5)	173.
O2—H2A···O9^v^	0.82	1.73	2.535 (5)	168.
N4—H4···O7^vi^	0.86	1.94	2.787 (6)	166.
N3—H3···O6	0.86	2.01	2.746 (6)	143.
N2—H2···O3	0.86	1.98	2.810 (5)	163.
N1—H1···O10	0.86	2.22	2.900 (6)	136.
N1—H1···O1	0.86	2.05	2.697 (4)	131.
C4—H4A···O5^vii^	0.93	2.34	3.162 (7)	147
C6—H6···O3^iv^	0.93	2.26	3.161 (6)	162
C11—H11···O8^vi^	0.93	2.57	3.252 (8)	131
C15—H15A···O3^ii^	0.96	2.58	3.533 (7)	174

Symmetry codes: (i) *x*−1, *y*, *z*; (ii) −*x*+1/2, *y*−1/2, *z*; (iii) −*x*+3/2, *y*−1/2, *z*; (iv) *x*+1, *y*, *z*; (v) −*x*+1, −*y*+1, −*z*+1; (vi) *x*−1/2, *y*, −*z*+3/2; (vii) *x*+1/2, −*y*+3/2, −*z*+1.

## References

[bb1] Biswas, C., Drew, M. G. B., Escudero, D., Frontera, A. & Ghosh, A. (2009). *Eur. J. Inorg. Chem.* pp. 2238–2246.

[bb2] Bruker (2002). *SMART*, *SAINT* and *SADABS* Bruker AXS Inc., Madison, Wisconsin, USA.

[bb3] Desiraju, G. R. (2002). *Acc. Chem. Res.* **35**, 565–573. 10.1021/ar010054t 12118996

[bb4] Jin, S. W., Zhang, W. B., Liu, L., Gao, H. F., Wang, D. Q., Chen, R. P. & Xu, X. L. (2010). *J. Mol. Struct.* **975**, 128–136.

[bb5] Lam, C. K. & Mak, T. C. W. (2000). *Tetrahedron*, **56**, 6657–6665.

[bb6] Liu, J. Q., Wang, Y. Y., Ma, L. F., Zhang, W. H., Zeng, X. R., Zhong, F., Shi, Q. Z. & Peng, S. M. (2008). *Inorg. Chim. Acta*, **361**, 173–182.

[bb7] Sheldrick, G. M. (2008). *Acta Cryst.* A**64**, 112–122. 10.1107/S0108767307043930 18156677

